# Comparative Genomic Analysis of the Pattern of Evolution of Male and Female Reproductive Proteins in Seed Beetles

**DOI:** 10.1093/gbe/evae143

**Published:** 2024-06-28

**Authors:** Konstantinos Papachristos, Ahmed Sayadi, Göran Arnqvist

**Affiliations:** Molecular Evolution, Department of Cell and Molecular Biology, Uppsala University, Uppsala, Sweden; Rheumatology, Department of Medical Sciences, Uppsala University, Uppsala, Sweden; Animal Ecology, Department of Ecology and Genetics, Uppsala University, Uppsala, Sweden

**Keywords:** seminal fluid proteins, speciation, coevolution, sexual selection, sperm competition, Bruchinae

## Abstract

Male seminal fluid proteins often show signs of positive selection and divergent evolution, believed to reflect male–female coevolution. Yet, our understanding of the predicted concerted evolution of seminal fluid proteins and female reproductive proteins is limited. We sequenced, assembled, and annotated the genome of two species of seed beetles allowing a comparative analysis of four closely related species of these herbivorous insects. We compare the general pattern of evolution in genes encoding seminal fluid proteins and female reproductive proteins with those in digestive protein genes and well-conserved reference genes. We found that female reproductive proteins showed an overall ratio of nonsynonymous to synonymous substitutions (ω) similar to that of conserved genes, while seminal fluid proteins and digestive proteins exhibited higher overall ω values. Further, seminal fluid proteins and digestive proteins showed a higher proportion of sites putatively under positive selection, and explicit tests showed no difference in relaxed selection between protein types. Evolutionary rate covariation analyses showed that evolutionary rates among seminal fluid proteins were on average more closely correlated with those in female reproductive proteins than with either digestive or conserved genes. Gene expression showed the expected negative covariation with ω values, except for male-biased genes where this negative relationship was reversed. In conclusion, seminal fluid proteins showed relatively rapid evolution and signs of positive selection. In contrast, female reproductive proteins evolved at a lower rate under selective constraints, on par with genes known to be well conserved. Although our findings provide support for concerted evolution of seminal fluid proteins and female reproductive proteins, they also suggest that these two classes of proteins evolve under partly distinct selective regimes.

**Significance**

Genes encoding male reproductive proteins evolve rapidly in many animals, but much less is known about the evolution of those female reproductive proteins that male proteins are thought to interact with. We show that male reproductive proteins evolve relatively rapidly in a group of beetles, while female reproductive proteins evolve at a considerably lower rate. Yet, we find that correlated evolution between male and female reproductive proteins is more pronounced than between others groups of proteins, consistent with the hypothesis that these two classes of proteins are coevolving.

## Introduction

Several processes can in theory generate divergence between lineages, ultimately resulting in reproductive isolation and speciation, but determining their relative importance has proven to be very difficult (Schluter 2009; Servedio and Boughman 2017). The fact that closely related species are often more different from one another in sexual traits (e.g. Arnqvist 1998; Cooney et al. 2019) and reproductive genes (e.g. Swanson et al. 2001; Walters and Harrison 2010; Rowe et al. 2020) than in other types of traits and genes has spotlighted reproductive interactions between the sexes and sexual selection as an important driver of divergence (Mendelson and Safran 2021). Insights offered by genomic data in this field primarily relate to genes that encode male seminal fluid proteins (SFPs) that are transferred to females during mating. These proteins mediate a series of reproductive processes in females, affect male competitive fertilization success, tend to evolve rapidly and some show hallmarks of positive selection in diverse taxa (e.g. Swanson et al. 2001; Swanson and Vacquier 2002; Avila et al. 2011; Ramm 2020; Rowe et al. 2020; Wigby et al. 2020; Patlar et al. 2021; Wiberg et al. 2021).

While many SFPs have effects on male–male interactions and sperm competition success (e.g. Avila et al. 2011; Ramm 2020), their divergent evolution is also believed to be affected by male–female interactions and coevolution involving SFPs and female reproductive proteins (FRPs) (Arnqvist 2006; Manier et al. 2013; Sirot et al. 2015; Chapman 2018; Gasparini et al. 2020). Here, evolution of some FRPs would exert selection on and spur evolution of those SFPs that interact with FRPs in determining the outcome of mating interactions, in particular, male competitive fertilization success and female fecundity. In this manner, postmating sexual selection is hypothesized to generate rapid concerted evolution of a subset of SFPs and FRPs (Rice 1996; Gasparini et al. 2020). Yet, although females are known to express a multitude of FRPs in their reproductive tract in a variety taxa (Yapici et al. 2008; Meslin et al. 2015; Bayram et al. 2019; Dosselli et al. 2019; Plakke et al. 2019; McCullough et al. 2020; Veltsos et al. 2022), FRPs have not been characterized in most taxa, and our understanding of their evolution is relatively limited (Sirot et al. 2015). A few FRPs do show signs of positive selection in mammals (Swanson et al. 2001), and a subset of FRPs show signs of positive selection and rapid evolution in *Drosophila* (Swanson et al. 2004; Panhuis and Swanson 2006; Findlay et al. 2014; McDonough-Goldstein et al. 2021). However, the general pattern of evolution of FRPs is currently unclear, and we are aware of only one direct test of the key prediction of correlated evolution between SFPs and FRPs. Using Evolutionary Rate Covariation (ERC) analyses across *Drosophila* species, Findlay et al. (2014) identified 3 (out of 226) FRPs that showed apparent correlated evolution with a set of SFPs, although the overall correlation between the two classes of proteins was rather low. While FRPs may prove to generally show rapid divergent evolution as a result of male–female coevolution, there are also reasons to believe that FRPs might instead often experience relatively high selective constraints. Genes with female-biased expression are known to show relative low rates of evolution in *Drosophila* (Zhang et al. 2007), and this has been ascribed to a relatively high degree of pleiotropy among female-biased genes (Dean and Mank 2016; Rowe et al. 2018), a pattern also documented in FRPs (Meslin et al. 2015; Bayram et al. 2019). This is expected to result in a higher degree of functional and evolutionary constraints among FRPs.

Here, we employ a comparative genomic approach to assess the overall pattern of evolution of SFPs and FRPs in a group of insects where these patterns are unknown. Seed beetles (Coleoptera; Bruchinae) are granivorous insects, including some species which are major agricultural pests, which are an emerging model system in ecology and evolution. In terms of their reproductive biology, female seed beetles mate multiply (Katvala et al. 2008), males transfer a large ejaculate (Arnqvist et al. 2022), and postmating sexual selection is pronounced (e.g. Wilson et al. 1997; Brown and Eady 2001; Hotzy and Arnqvist 2009; Fritzsche and Arnqvist 2013). In one species (*Callosobruchus maculatus*), previous proteomic and transcriptomic efforts using whole-organism stable isotope labeling identified 317 proteins that were transferred by males to females at mating and 231 female-derived proteins that are upregulated in the female reproductive tract following mating (Bayram et al. 2017, 2019). Moreover, the composition of SFPs in the ejaculate has direct effects on male competitive fertilization success in seed beetles (Goenaga et al. 2015; Yamane et al. 2015), and some classes of SFPs are beneficial for females (Savalli and Fox 1999; Takakura 2006; Yamane et al. 2015; Arnqvist et al. 2022), who digest the ejaculate in their reproductive tract (Huignard 1983; Takakura 2006; Arnqvist et al. 2022), while others have toxic effects (Das et al. 1980; Yamane 2013). Thus, phenotypic studies show that there is a potential for rapid evolution of reproductive proteins and of SFP–FRP coevolution in this group of insects, fueled by sexual selection (Arnqvist and Rowe 2005; Gasparini et al. 2020). A documentation of conspecific sperm precedence in seed beetles also implies that reproductive proteins may be involved in incipient speciation (Rugman-Jones and Eady 2007). Here, we sequenced and de novo assembled the genomes of *Callosobruchus chinensis* and *Callosobruchus analis* which then enabled the first comparative genomic analyses of SFP and FRP orthologs in four species of seed beetles, given that genome assemblies are already available for two species (*C. maculatus* and *Acanthoscelides obtectus*) (Sayadi et al. 2019; Immonen et al. 2023; Arnqvist et al. 2024).

Our aim here is to compare the overall pattern of evolution across four classes of proteins. These are SFPs, FRPs, and two additional classes of proteins. First, we use a set of genes previously annotated as digestive enzymes in *C. maculatus* (Sayadi et al. 2019). The legume host seeds of these insects contain large amounts of secondary compounds that serve as a defense against seed predators (Wink 2013), and seed beetle larvae produce a diverse set of enzymes in their gut that allow them to detoxify and digest the host seeds (Zhu-Salzman and Zeng 2015). Different beetle species utilize different legume hosts with a unique composition of defensive compounds. Digestive enzymes generally evolve relatively rapidly among herbivorous insects and show signs of positive selection, as changes in their amino acid sequences permit the detoxification of distinct secondary metabolites during host shifts (Simon et al. 2015). Across seed beetles, we thus expect these proteins to show relatively high rates of evolution and signs of positive selection (Cohen et al. 2021) since the four species used here differ in their use of legume hosts (Udayagiri and Wadhi 1989). Second, we used a set of single-copy Busco genes (see SI) of *C. maculatus* known to be well conserved across all arthropods. These genes are expected to show relatively low rates of evolution and high selective constraints (Simão et al. 2015).

Here, we first model the rate of synonymous and nonsynonymous substitutions (dN/dS) across species for these classes of genes, test for overall signs of positive and relaxed selection, and then assess overall correlated evolution using an ERC (Clark et al. 2012) approach. Finally, we ask whether and how sex-biased gene expression affects the rate of evolution across gene classes, as sex-limited expression may lead to relaxed selection and affect the rate of evolution of reproductive proteins (Dapper and Wade 2016).

## Results

**Fig. 1.**
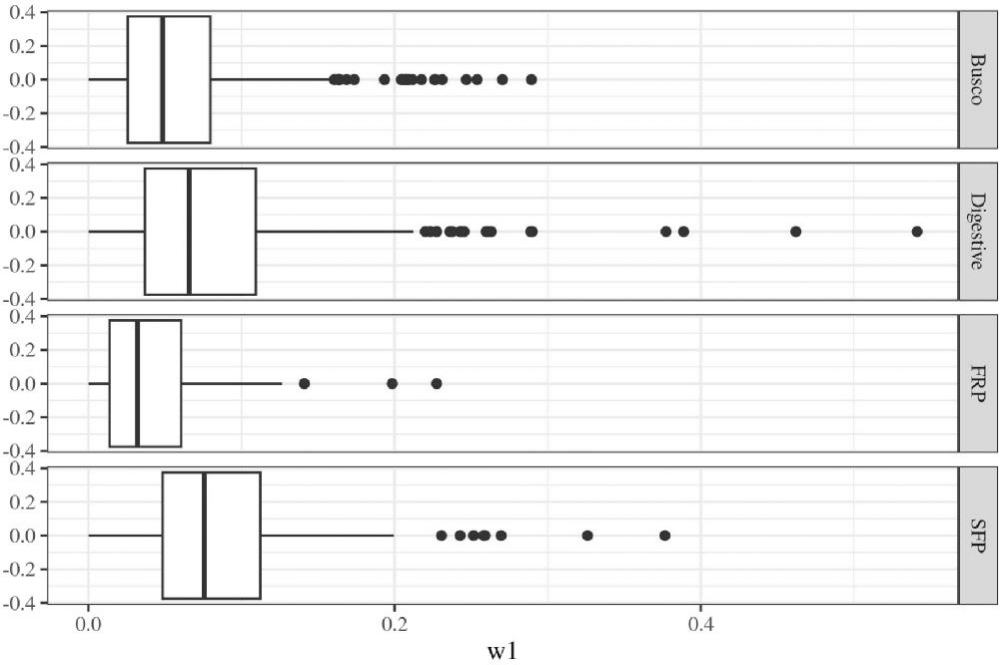
Box plots of gene-specific overall ω across all proteins, derived from M0 models. Vertical lines represent medians.

**Fig. 2.**
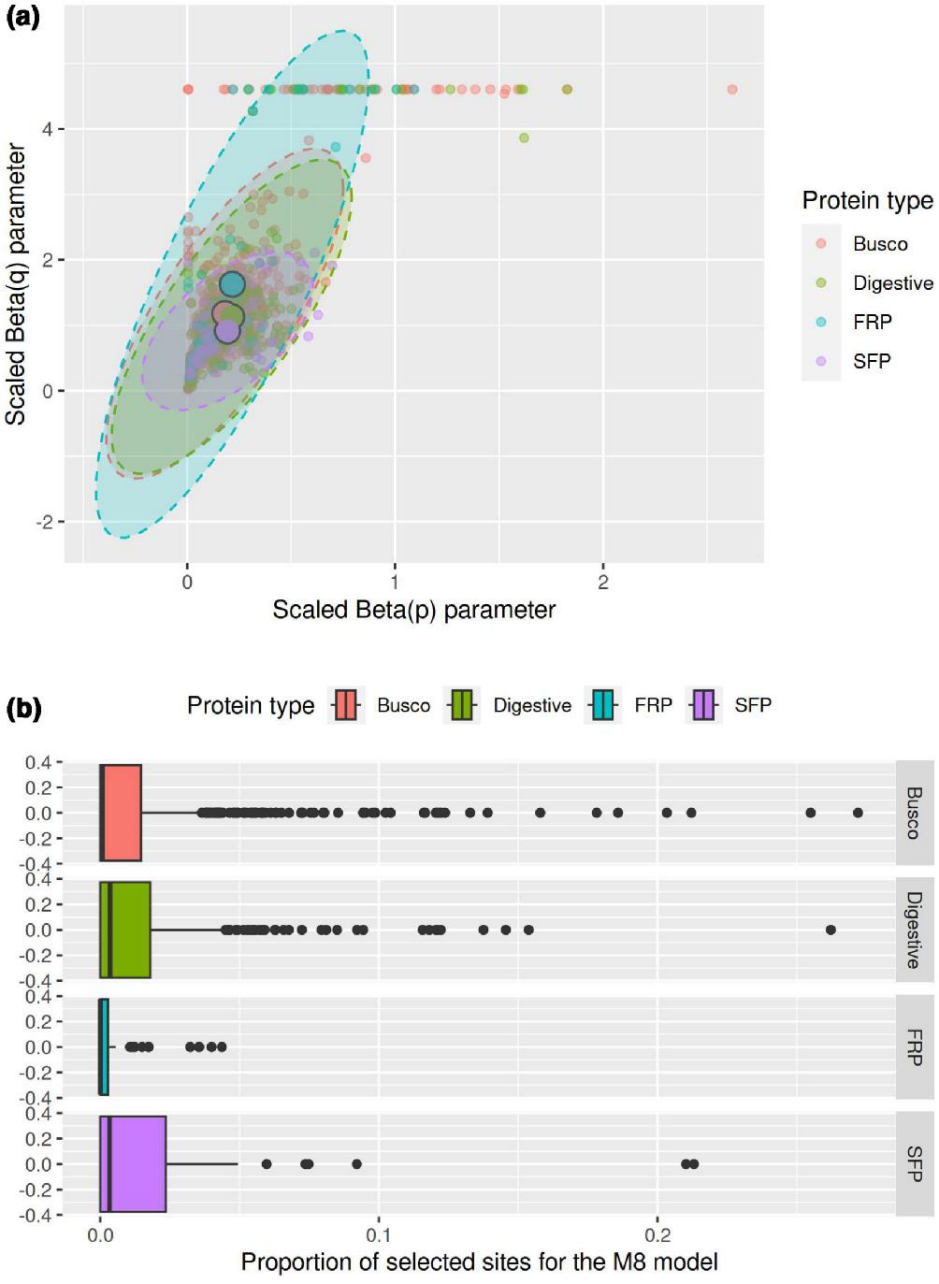
a) The distribution of the shape parameters (log_10_  *p* and log_10_  *q*) of the beta distribution of ω values across all proteins, derived from M7 models. b) Box plots of the proportion of sites showing ω > 1 across all proteins, derived from M8 models.

Our models of ω across genes showed that the four classes of proteins overall show somewhat different signals of selection. The simplest model (M0) assumes that all sites of proteins of all species evolve under one common ω value, and the distribution of estimated ω values for this model across proteins is illustrated in Fig. 1. Pairwise comparison between these distributions, using Kolmogorov–Smirnov tests, showed that while busco proteins and FRPs did not differ significantly from one another (*P* = 0.41), all other classes of proteins did (*P* < 0.01 in all cases). In general, SFPs and digestive proteins showed the highest gene-specific ω and busco and FRPs the lowest (Fig. 1), consistent with a relatively more prominent role for purifying selection in busco proteins and FRPs. The M7 site models allow ω to vary from 0 to 1 across sites in a given gene, by fitting ω across sites as a beta distribution with the two shape parameters *p* and *q*. The shape of the beta distribution should thus vary across sets of proteins that differ in selective regimes. Figure 2a shows the distribution of the shape parameters across protein sets. A linear model of log_10_ (1 + *q*), using log_10_ (1 + *p*) and protein type as predictors, showed that the overall shape of the beta distribution of ω in M7 models differed across protein types (protein type: *F*_3,1244_ = 12.23, *P* < 0.001; protein type × *p*: *F*_3,1244_ = 10.18, *P* < 0.001) and was most skewed toward low values of ω among FRPs and least so in SFPs. The M8 model also fits a beta distribution but in addition allows for a proportion of sites to show ω > 1 (i.e. signs of positive selection) and thus to fall outside of the beta distribution. This proportion differed significantly across gene sets (Kruskal–Wallis χ^2^ = 15.6, degrees of freedom [df] = 3, *P* = 0.001) and was highest overall in SFPs and digestive proteins (Fig. 2b).

Although the overall dN/dS ratios thus varied across protein types, few single genes passed explicit log-likelihood ratio tests (LRT; following false discovery rate [FDR] compensation) for positive selection. The percentage (%) of genes passing the three model comparisons (M1a–M2a, M7–M8, and M8–M8a) were as follows: Busco: 1.39, 3.41, and 2.15; digestive: 1.78, 4.15, and 2.97; FRPs: 1.82, 3.64, and 1.82; and SFPs: 0, 2.94, and 0. We note that the power of these FDR-compensated tests is low, due to a restricted number of species and a large number of genes (Álvarez-Carretero et al. 2023).

The relatively high incidence of high values of ω among SFPs and digestive proteins could be due to a larger influence of positive selection (ω > 1) but could, alternatively, be due to relaxed selection (ω = 1). However, our RELAX analyses provided no evidence for a larger influence of relaxed selection in these protein types. Overall, the highest proportion of tests showing significant (*q* < 0.05) intensified selection (i.e. *k* > 1) was SFPs (2.9%), and the lowest was FRPs (0.9%), but the proportion did not vary significantly across the four protein types (Fisher's exact test: *P* = 0.288; supplementary fig. S3, Supplementary Material online). More importantly, few genes showed significant relaxed selection, and the highest proportion of tests showing significant relaxed selection (i.e. *k* < 1) was digestive proteins (1.7%), and the lowest was FRPs (0.5%), but the proportion again did not differ significantly across protein types (Fisher's exact test: *P* = 0.569).

The overall distribution of neutrality index (NI) differed significantly across the four proteins types (Anderson–Darling test; *P* = 0.047), but relatively few genes showed NI < 1 (Fisher's exact tests; *q* = 0.05), indicative of positive selection. Although the proportion of genes that showed a NI significantly <1 in these McDonald–Kreitman (MK) tests was highest for SFPs, this proportion (Busco: 4.3%, digestive: 4.4%, FRPs: 3.7%, and SFPs: 7.4%) did not differ significantly across protein types (Fisher's exact test: *P* = 0.644).

**Fig. 3.**
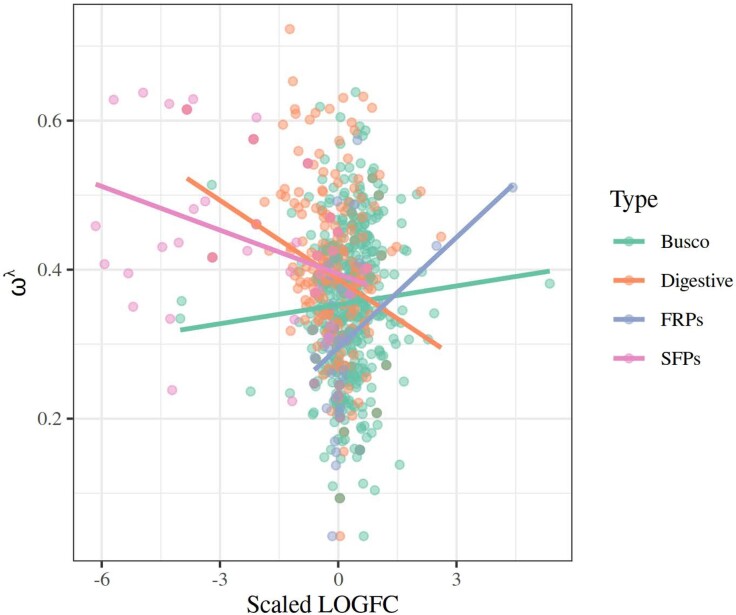
The relationship between sex bias in gene expression and ω differed significantly across protein sets. Here, genes with negative LogFC values are male-biased, and those with positive values are female-biased. Interestingly, more sex-biased expression of reproductive proteins (both SFPs and FRPs) showed higher ω values.

**Fig. 4.**
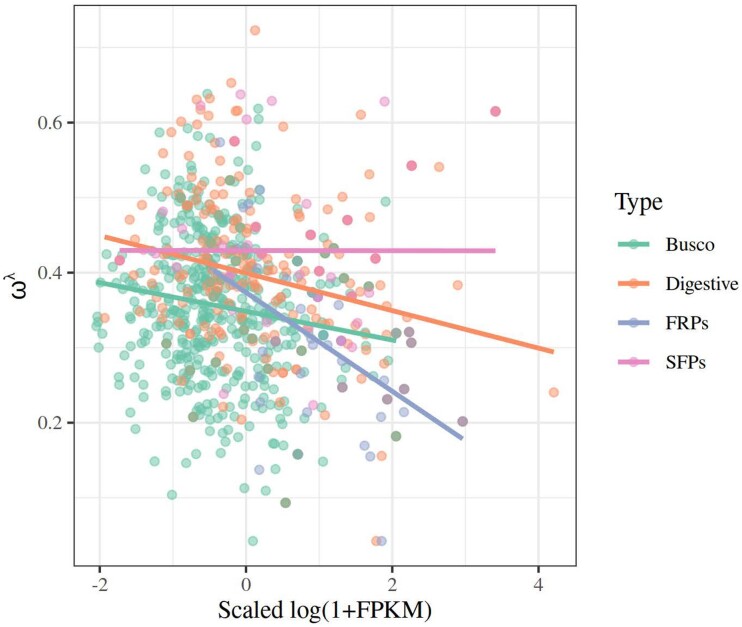
The relationship between gene expression (log FPKM) and ω differed significantly across protein sets. While this relationship was negative for digestive, BUSCO, and FRP genes, it was not for SFPs, reflecting the fact that some highly expressed SFPs showed relatively high ω values.

**Fig. 5.**
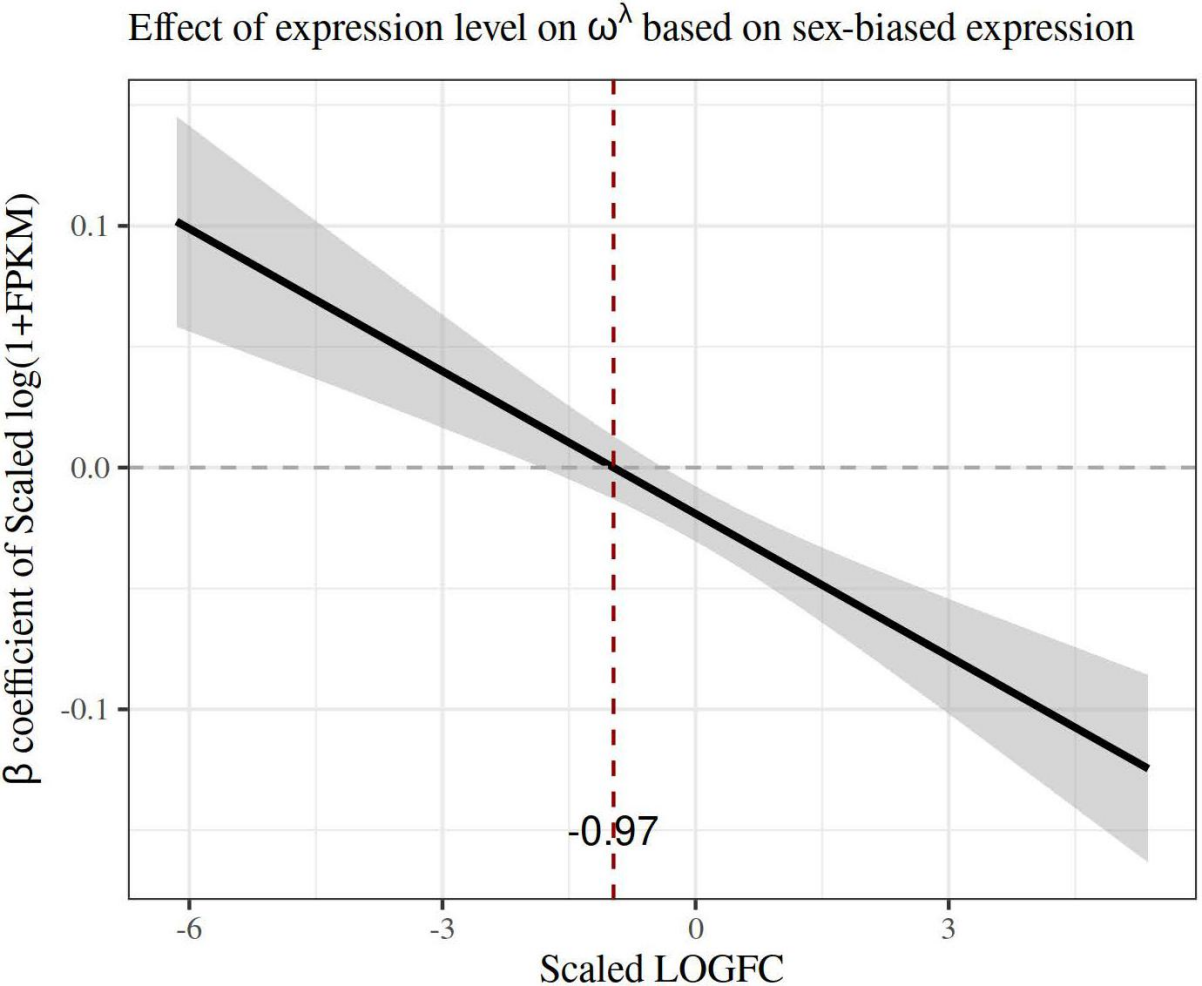
The relationship between overall gene expression and ω is negative for genes with female-biased expression and those with unbiased expression but positive for genes with male-biased expression. This figure visualizes the interactive effect of FPKM:LogFC on ω (Table 1). It shows the predicted conditional effect of sex-biased expression (LogFC) along the abscissa on the slope (±95% CI) between overall gene expression (log FPKM) and ω, along the ordinate. Genes with negative LogFC values are male-biased, and those with positive values are female-biased.

In order to assess whether and how the pattern of gene expression affects the rate of evolution of proteins, we fitted a linear model using Box-Cox–transformed ω values (from M0 models) as our response variable. The predictors examined in these models were the overall expression level (fragments per kilobase of transcript per million of mapped reads [FPKM]) and sex bias in gene expression fold change converted to a logarithmic scale (LogFC) for each gene and protein type (factorial). We used extant data from *C. maculatus* for FPKM and LogFC (Immonen et al. 2017). We note that male SFPs were markedly male-biased in expression, more so than FRPs being female-biased (Fig. 3). The main effects of gene expression on ω and how this interacts with other variables are given in Table 1. Overall, high expression levels were significantly associated with low ω values, and the four types of proteins differ in overall rate of evolution also when statistically controlling for gene expression (Fig. 4). However, both of these effects were contingent upon other factors, evident from the sizeable and significant interaction terms (Table 1). There was a strong FPKM × LogFC interaction, such that genes with unbiased and female-biased expression showed the predicted negative expression-rate (E-R) correlation while genes with strongly male-biased expression instead showed a positive E-R correlation (Fig. 5). Further, the effects of overall and sex-biased expression differed across protein types. The E-R correlation was negative for digestive, busco and FRP genes, but this was not true for SFPs (Fig. 4). Moreover, increased sex bias was associated with elevated ω for both SFPs and FRPs but less so in busco genes (Fig. 3). In summary, this model revealed a classic negative E-R correlation overall but also showed that male-biased genes instead show a positive E-R relationship. Finally, we note that we replicated the above analyses using ω values not from M0 models but instead from the *C. maculatus* branch in free-ratio branch models. These analyses yielded results that were qualitatively identical and quantitatively very similar to those reported above (see supplementary fig. S1, Supplementary Material online).

**Table 1:** Linear model of the effects of gene expression (FPKM), sex bias in gene expression (LogFC), and protein type on ω across all proteins

Source	Type III SS	df	*F*	*P*
FPKM	0.1	1	10.74	0.001
LogFC	0.02	1	2.48	0.115
Type	0.37	3	13.56	<0.001
FPKM:LogFC	0.28	1	31.45	<0.001
FPKM:Type	0.09	3	3.42	0.016
LogFC:Type	0.2	3	7.32	<0.001
Error	7.6	843	…	…

**Fig. 6.**
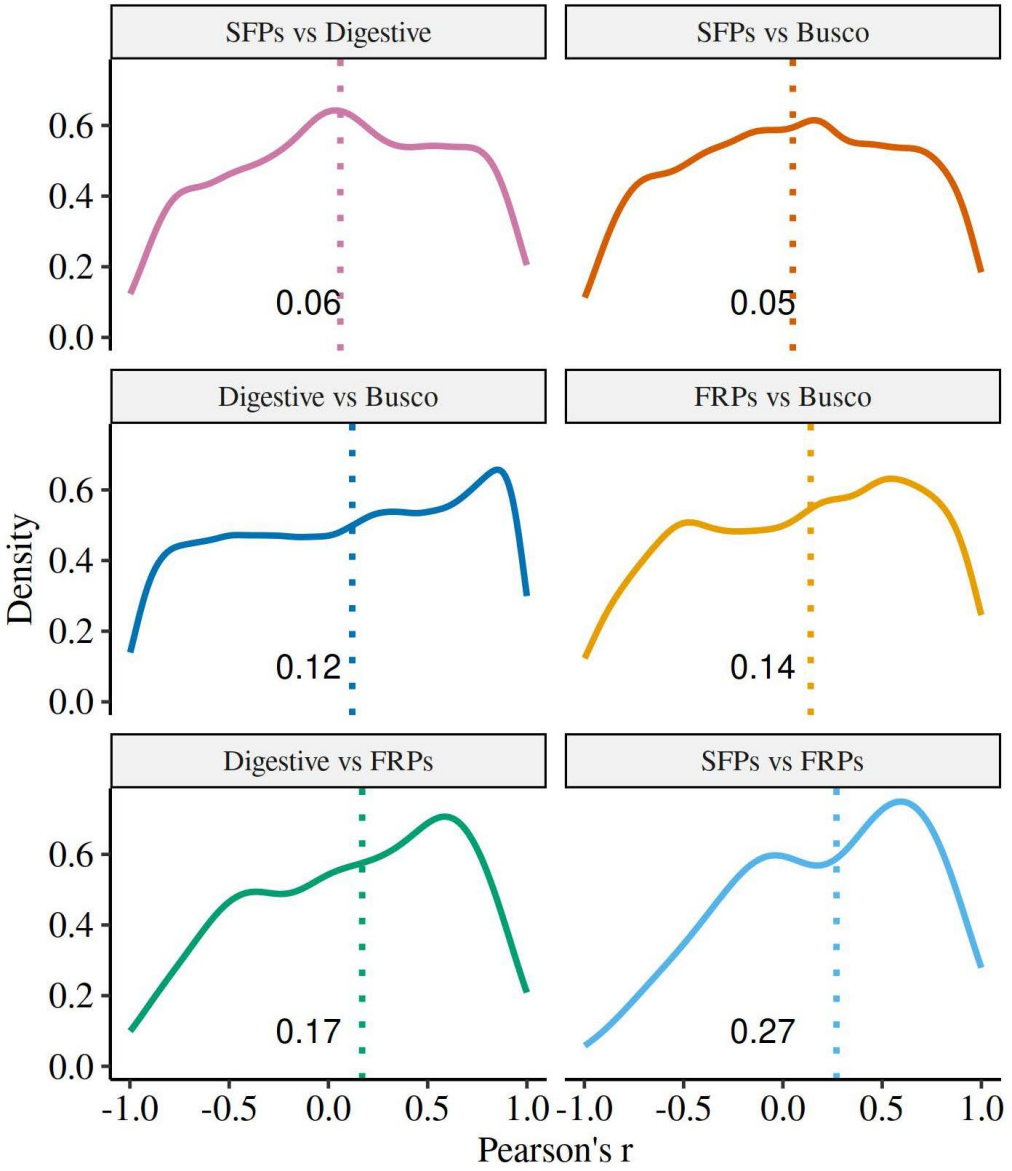
The distribution of correlation coefficients from correlations of branch-specific ω values between pair of proteins sets, based on the free-ratio model. This measure of overall ERC was stronger for SFPs and FRPs than for any other pair of protein sets. The value of the median for each distribution is indicated as is its position.

Our omnibus ERC analyses of the pattern of correlated evolution across gene sets are summarized in Fig. 6. The distribution of correlations across the six pairwise comparisons was highly significantly different (Kruskal–Wallis χ^2^ = 50.4, df = 5, *P* = 1.1 × 10^−9^), being highest overall between SFPs and FRPs and lowest between SFPs and digestive enzymes. We then asked which specific pairs of SFPs and FRPs that showed a significant correlation in ω across the phylogeny. However, because of the very large set of pairwise correlations (*N* = 8564), a FDR correction for multiple testing left only five significant (at *q* < 0.05) positive correlations, involving three different SFPs and five FRPs (supplementary table S4, Supplementary Material online).

## Discussion

This study set out to characterize the overall pattern of evolution of male and female reproductive proteins in seed beetles, an emerging model system in evolutionary genetics. By sequencing and de novo assembling the genomes of two additional species, we were able to assess whether the evolution of male and female reproductive proteins shows the rapid and correlated evolution predicted by theory. Our analyses generated three insights. First, we found that male SFPs in general showed a more rapid evolution than did FRPs. In fact, male SFPs evolved at a rate comparable with digestive proteins, known to experience selection and divergent evolution in this group of insects. Second, we found that the relationship between gene expression and the rate of evolution differed across sets of genes, which may offer novel insights into reproductive protein evolution. Finally, we found that the ratio of nonsynonymous to synonymous substitutions was more concerted between SFPs and FRPs than between other pairwise sets of proteins, consistent with a role for male–female coevolution. Below, we discuss each of these findings in more detail.

The general pattern of substitutions in SFPs was similar to that in digestive enzymes, a classic set of rapidly and divergently evolving functional proteins in herbivorous insects in general (e.g. Simon et al. 2015) and in leaf beetles more specifically (e.g. Cohen et al. 2021). The overall evolution of SFPs is consistent with a role for divergent postmating sexual selection affecting the evolution of these genes, as suggested by previous phenotypic work in seed beetles (Goenaga et al. 2015; Yamane et al. 2015). However, in theory, genes encoding SFPs will also experience relaxed selective constraints when their expression is sex-limited (Dapper and Wade 2016), and this may elevate evolutionary diversification of SFPs (Dapper and Wade 2020). Although relaxed selection could thus affect this class of genes, four observations suggest that relaxed selection is not the main cause for the overall pattern of evolution seen among SFPs here. First, the majority of the seed beetles’ SFPs included here are sex-biased rather than sex-limited in their expression. Although the precise delineation between sex bias and sex limitation based on transcript abundance data is somewhat ambiguous, only a handful of SFPs show expression in males >32 times as high as in females (i.e. LogFC < −5). These did not noticeably differ from less sex-biased SFPs, and overall sex bias in expression was not related to ω across genes (Table 1). Second, our explicit tests of relaxed selection provided no evidence for relaxed selection being more prevalent among SFPs. A few other studies have documented relaxed selection in a larger fraction of SFPs than we found here (e.g. Patlar et al. 2021). The reasons for this are unclear, but it may be related to the fact that postmating sexual selection is relatively strong in seed beetles (Fritzsche and Arnqvist 2013). Third, Dapper and Wade (2020) noted that metrics that use information on polymorphism within species, such as the MK tests, can better distinguish relaxed selection from positive selection. We found that the proportion of genes showing signs of positive selection in MK tests was highest in SFPs, although this proportion did not differ significantly across protein classes. Fourth, we note that a previous study of one of the species studied here, *C. maculatus*, found that SFPs contribute disproportionally to population differentiation but that the contribution to differentiation was not related to relaxed selection across SFP genes, inferred from within-population the ratio of nonsynonymous and synonymous polymorphism (Arnqvist and Sayadi 2022).

Our analysis provided support for a general negative relationship between gene expression and ω across genes, consistent with stronger purifying selection in highly expressed genes, but this E-R anticorrelation was interestingly enough nullified for SFPs and even reversed for strongly male-biased genes. Among male-biased genes, higher expression was instead associated with relatively high ω. The E-R anticorrelation is essentially thought to reflect stronger selective constraints in more highly expressed genes (Zhang and Yang 2015; Feyertag et al. 2017). It is difficult to envision why high expression per se would be associated with weaker selective constraints and a closer inspection showed that the E-R anticorrelation is not nullified for SFPs by highly expressed SFPs with a high ω showing signs of relaxed selection (supplementary fig. S2, Supplementary Material online). It is, however, possible that highly expressed male-biased genes are more likely to experience diversifying positive selection in some regions than those that are expressed at lower levels, simply because the former may play a more central role (i.e. have larger phenotypic effects) in sexual selection. This could nullify or even reverse the E-R anticorrelation. Although in no way conclusive, these findings thus lend novel support to the hypothesis that the evolution of male-biased SFPs is more affected by positive selection than the other protein classes investigated here.

Another observation also provides support for a rapid and divergent evolution of SFPs in this group. Bayram et al. (2019) noted that some 30% of all *C. maculatus* SFPs were entirely novel, showing no known homologs in extant databases. In the same vein, our search for gene orthologs of *C. maculatus* genes in the genomes of the other three species showed the lowest fraction of complete strict orthogroups for SFPs (21%), consistent with a rapid and divergent evolution of SFPs within this clade. Our results should perhaps be seen in light of the fact that, as in all comparative genomic studies, the most divergently evolving proteins will in fact not be included in the full analyses. This may underestimate the true relative rates of evolution.

As a class, FRPs showed a somewhat different pattern of evolution. Overall ω was lower than for SFPs, and the evolution of FRPs was more similar to that of conserved busco genes. A very similar pattern has been documented in *Drosophila* and butterflies (e.g. Zhang et al. 2007; Meslin et al. 2015). A more rapid evolution of reproductive phenotypic traits in males than in females is a fairly general pattern (Hedrick and Temeles 1989; Andersson 1994), reflecting asymmetries in sexual selection between the sexes (Arnqvist 2006; Janicke et al. 2016; Winkler et al. 2021). For reproductive proteins, this pattern has been ascribed to (i) more rigid evolutionary constraints among FRPs due to a higher degree of pleiotropy, (ii) relatively minor genetic modifications of FRPs generating larger evolutionary responses in SFPs or (iii) a larger influence of additional sources of diversifying selection (e.g. sexual selection through sperm competition) in SFPs (Arnqvist 2006; Dean and Mank 2016; Rowe et al. 2018). In the beetles studied here, higher pleiotropy in FRPs is supported by the pattern of gene expression in *C. maculatus*. For example, the overlap between female-biased genes of the abdomen with those in the head and the thorax is higher than for male-biased genes (Immonen et al. 2017), and FRPs show a lower degree of sex-biased expression than do SFPs (Bayram et al. 2019). Both of these observations suggest that FRP genes are more pleiotropic than genes encoding SFPs. In addition, population comparisons have shown that while populations of *C. maculatus* have diverged more in SFPs than in other genes, this is not true for FRPs (Arnqvist and Sayadi 2022). Yet, we found that a few FRPs did show signs of positive selection. It is interesting to note that the FRPs of *C. maculatus* is known to contain a large number proteases and the SFPs includes a number of protease inhibitors (Bayram et al. 2019). The latter are thought to protect sperm from proteolytic attack in the female reproductive tract (Dean et al. 2009), consistent with the suggested antagonistic interactions between female-derived proteases and male-derived protease inhibitors (Sirot et al. 2015) which may generate positive reciprocal selection for novelty in both sets of proteins.

Our ERC analyses showed that the highest rate of overall correlated evolution across proteins sets occurred between SFPs and FRPs. This is broadly consistent with concerted coevolution of these two classes of proteins, as the strength of postmating sexual selection may have varied across branches in the evolutionary history of these species (Arnqvist et al. 2022). Here, functional interactions between male and female proteins would then have selected for novelty in these interacting classes of proteins (Sirot et al. 2015). However, these inferences are based on correlational data, and even if the pattern is consistent with predictions, it is important to acknowledge that a pattern of correlated evolution can be generated in several ways other than coevolution. This includes, for example, shared evolutionary constraints (Hakes et al. 2007) or a shared dependency on a third causal factor or set of proteins.

## Conclusions

Overall, our findings show that SFPs evolved at a rate which was higher than in FRPs, and we show that signs of positive selection were more frequent in SFPs than in FRPs. In fact, the overall rate of evolution of FRPs was comparable with busco genes, known to be conserved and under selective constraints. Yet, correlated evolution between SFPs and FRPs was elevated compared with that between other sets of proteins. The fact that male SFPs showed a more rapid evolution is consistent with the propositions that FRPs experience higher pleiotropic constraints and that relatively minor modifications of FRPs can generate continual and strong sexual selection for novelty among SFPs (Arnqvist and Rowe 2005; Rowe et al. 2018). We note that several female-derived proteins other than those expressed in the female reproductive tract (e.g. neuroreceptor proteins) are known to interact with SFPs (Sirot et al. 2015), so it certainly remains possible that some rapidly evolving proteins which interact with SFPs have not been included in our analyses.

Our inferences are constrained by the fact that only four species are included in this study. However, including more species would no doubt further have limited the number of proteins actually included in our analyses, simply because the number of complete sets of strict orthologs is reduced when more species are included when focal genes are evolving rapidly. Finally, we note that our data and analyses are, inevitably, correlational. It is therefore difficult to make firm conclusions beyond the overall pattern of evolution of the protein sets investigated. Inferences regarding specific proteins and their interactions will need to rely on functional studies and improved annotations. We hope that the genomic resources and analyses we have presented will provide a basis for such work in the future in this emerging model system in reproductive biology and evolutionary genetics.

## Materials and Methods

### Genomic Resources

We employed the published genome assemblies of *C. maculatus* (Sayadi et al. 2019) and *A. obtectus* (Immonen et al. 2023; European Nucleotide Archive, accessions PRJEB30475 and PRJEB51445, respectively). Both assemblies are well-annotated high-quality assemblies based on long-read PacBio sequencing data. In addition, we sequenced, de novo assembled and annotated the genomes of *C. chinensis* and *C. analis*. Briefly, for both species, PacBio long-read sequences representing ≈60× genomic coverage with an average read length of ≈10 kb were assembled using FALCON. The resulting two polished genome assemblies (European Nucleotide Archive, accessions PRJEB70760 and PRJEB70763) are 701 and 959 Mb in total size (estimated fraction of genome covered by assembly 97.7% and 98.8%), with an N50 of 800 and 240 kb. We employed a comprehensive MAKER3 annotation pipeline using transcriptome data from closely related species, homology, and ab initio prediction methods and identified 35,426 and 33,285 coding genes, respectively. Despite a high repeat content in the assemblies, evaluations based on conserved proteins sets showed a high fraction of well-assembled genes in the assemblies (busco: 98.1% and 92.1% complete, respectively). We refer to the SI for details on the sequencing, assembly, annotation, and evaluation of the two genomes.

### Protein Sets and Orthologous Genes

In a previous study of *C. maculatus*, Bayram et al. (2019) used males and females that were labeled with stable isotopes to infer SFPs and FRPs based on a transcriptomic and proteomic approach (Findlay et al. 2008). They identified 317 male-derived proteins that were transferred to females at mating, and these proteins form our SFP set. They also identified 231 female-derived proteins that are expressed in the female reproductive tract and are upregulated following mating. These proteins form our FRP set. Several previous studies of gene expression in the midgut of *C. maculatus* larvae (see Sayadi et al. 2019) have identified 741 enzymes and other proteins that collectively form the digestive machinery allowing growing larvae to detoxify, digest, and metabolize the host seeds. These digestive proteins form our third set. Our final protein set represents 1,137 genes, present in single-copy orthologs in the genome of *C. maculatus*, from the core busco gene set for Arthropoda. These busco proteins constitute our fourth set.

Hierarchical orthogroups were identified among the four proteomes using Orthofinder version 2.4 (Emms and Kelly 2019). Nucleotide coding sequences for the proteome were translated into amino acid sequences using the program transeq of EM-BOSS v6.6.0.0 (Rice et al. 2000) and hierarchical orthogroups containing gene IDs of *C. maculatus* in any of the gene sets described above were extracted. Genes in each orthogroup were aligned and gene trees created. The hierarchical orthogroups were then converted to strict orthogroups and filtered using phylopypruner 0.9.7 (Thalén 2018). This yielded 68, 55, 337, and 792 complete strict orthogroups for the SFPs, FRPs, digestive proteins, and busco proteins, respectively. These were used for downstream analyses (see SI for details on the identification of orthologous genes).

### Evolutionary Modeling

*Callosobruchus maculatus* and *C. analis* are closely related sister species that diverged approximately 5 Ma (Tuda et al. 2006), while *C. chinensis* diverged from these approximately 22 Ma and *A. obtectus* diverged from the three *Callosobruchus* species some 45 Ma (Kergoat et al. 2005). To model evolution, we used a phylogeny representing the pruned supertree of Kergoat et al. (2008), resulting from 15 source trees based on large amounts of mitochondrial and nuclear genetic data as well as morphology (supplementary fig. S4, Supplementary Material online).

To model overall substitution rates, dN/dS ratios (i.e. ω), and the footprints of selection, we used three inferential routes. First, we used codeML 4.8a of the PAML package (Yang 2007), as implemented in the ETE3 python framework (Huerta-Cepas et al. 2016), which uses maximum likelihood methods to estimate ω. This involved first fitting M0 codon substitution models to provide overall gene-specific values of ω. To assess and explicitly test for the footprints of positive selection in codeML, we fitted the following site models in codeML, allowing ω to vary along the sequence but assuming a common ω among branches: M1a (nearly neutral), M2a (positive selection), M7 (beta), M8 (beta and *ω* > 1), and M8a (beta and *ω* = 1). The fit of these models to our sequence data was done by FDR-compensated LRTs based on the following model comparisons: M1a–M2a, M7–M8, and M8a–M8 (Álvarez-Carretero et al. 2023). Those genes passing the M1a–M2a comparison and those passing the M7–M8 and the M8–M8a comparisons were deemed showing significant signs of positive selection in this modeling effort.

Second, we employed the HyPhy v2.5.8 module RELAX (Wertheim et al. 2015) to explicitly test for relaxed selection. RELAX fits branch-site models including a parameter *k*, interpreted as a selection intensity parameter (Wertheim et al. 2015; Wiberg et al. 2021), which is then compared with a null model where *k* = 1 using a LRT. A significant model with *k* > 1 indicates that selection strength has been intensified along the foreground branches and a significant model with *k* < 1 indicates that selection has been relaxed. For each gene, we fitted four models using either of the four species as the foreground branch and those genes showing at least one significant deviation (following FDR compensation) from a model with *k* = 1 were deemed as showing significant signs of either relaxed or intensified (positive/purifying) selection.

Third, to further test for signs of positive selection, we conducted MK tests (McDonald and Kreitman 1991). In these neutrality tests, the numbers of nonsynonymous to synonymous substitutions between species are compared with the numbers of nonsynonymous and synonymous polymorphisms within populations (McDonald 2014). The NI (Rand and Kann 1996) is expected to be NI = 1 under neutral evolution, NI < 1 under positive selection, and NI > 1 under negative or balancing selection. In these MK tests, we retrieved polymorphism data from a whole-genome resequencing study of a population of *C. maculatus* (Brazil; Sayadi et al. 2019) as our source for data on pN and pS (see SI for details).

Data were imported in the R software environment for further statistical modeling and visualization (R Core Team 2022) and managed with the tidyverse package (Wickham et al. 2019). Proteins passing LRT for positive selection were annotated by InterProScan v.5.51-85.0 (Blum et al. 2021).

To assess potential overall footprints of coevolution between protein sets, we employed an ERC (Clark et al. 2012) approach. Branch-specific estimates of ω (estimated from free-ratio models) were correlated with one another, across all genes in pairs of gene sets, using the corrr package (Kuhn et al. 2022). Here, the prediction is that protein classes with members that interact, directly or indirectly, and coevolve should on average show reciprocal substitution patterns across the phylogeny resulting in higher correlations on average (Lovell and Robertson 2010). All ω values were log-transformed prior to analyses to normalize their distribution.

Finally, because differences in rates of evolution between genes and gene classes are often negatively related to their absolute expression (Yang et al. 2012), a common pattern known as the E-R anticorrelation (Zhang and Yang 2015), and may be affected by sex limitation in expression (Dapper and Wade 2016), we evaluated the role of variation in gene expression for variance in ω across genes using linear modeling. The purpose of this effort was two-fold. First, we wished to compare the rate of evolution across proteins types when removing potentially confounding variation due to absolute expression. Second, because the E-R anticorrelation is thought to reflect stronger purifying selection in highly expressed genes (Zhang and Yang 2015; Feyertag et al. 2017), we wished to assess whether the E-R anticorrelation manifests also in SPFs—a group of proteins hypothesized to more frequently experience diversifying selection. Here, we used data for absolute gene expression (FPKM) and sex-biased gene expression (LogFC) from a study of gene expression in the abdomen of *C. maculatus* (Immonen et al. 2017) as predictors. These analyses thus rely on the assumption that variation in gene expression in *C. maculatus* to some extent predicts variation in expression of orthologs in the other related species. We note that the cross-species correlation of mRNA transcript abundance of orthologous genes is typically sizeable between both distantly (e.g. Laurent et al. 2010) and more closely related taxa (e.g. Zhang et al. 2007; Stadler and Fire 2013). Data on FPKM and LogFC were standardized to zero mean and unit variance prior to fitting these models (see SI for details).

## Supplementary Material

evae143_Supplementary_Data

## Data Availability

The annotated genome assemblies, along with sequence data, for *C. chinensis* and *C. analis* are available from the European Nucleotide Archive under the project ID PRJEB70760 for *C. chinensis* and the project ID PRJEB70763 for *C. analis*.
