# Supplementary material for: Comparative Genomic Analysis of the Pattern of Evolution of Male and Female Reproductive Proteins in Seed Beetles
Source: Genome Biol Evol. 2024 Jun 28;16(7):evae143. doi: 10.1093/gbe/evae143 (PMC11251426; doi:10.1093/gbe/evae143)
Supplement: evae143_Supplementary_Data [file evae143_supplementary_data.pdf]

Molecular Biology and Evolution

Comparative genomic analyses of the pattern of evolution of male and female reproductive proteins in seed beetles

Konstantinos Papachristos, Ahmed Sayadi and Göran Arnqvist

Supplemental Information

1. Genome sequencing and assembly
2. Genome annotation
3. Orthogroup clustering
4. Estimation of dN:dS ratios
5. Evolutionary Rate Covariation
6. McDonald – Kreitman tests
7. Linear models
8. Phylogeny
9. References

1. Genome sequencing and assembly

We de novo sequenced, assembled and annotated the genomes of *Callosobruchus chinensis* and *C. analis*. We first subjected laboratory lines of both species to five consecutive generations of inbreeding by propagating a single female mated to a full sib brother. Males of inbred line per species were subsequently used for sequencing.

Samples of whole-body genomic high-molecular-weight DNA was extracted (10 males per sample) and submitted to long-read sequencing using PacBio. Extractions were made using QIAGEN Genomic-tip 20/G, according to the manufacturer's protocol. High molecular weight DNA was sheared using the Megaruptor 2 system (Diagenode) with a 20 kb target. Size-selection with a 8.5 kb cut-off was done using the Blue Pippin system (SAGE). SMRTbell Template Prep Kit 1.0 was used for library construction according to manufacturer's instructions. Sequencing was performed using 48 (*C. chinensis*) and 56 (*C. analis*) SMRT cells, respectively, on a RSII system, with 240 min movies and P6 chemistry.

See Table S1 for sequencing yield. The genome was then assembled using FALCON v 0.5.0 (https://github.com/PacificBiosciences/FALCON/) with default parameters, based on the PacBio read data. The assembly was subsequently error-corrected by one round of Quiver (SMART portal) based on re-alignement of the full set of PacBio reads.

Table S1: PacBio sequence read data.

	C. chinensis	***C. analis***
Total number of reads	4,452,545	5,685,453
Average read-length	9,199	10,589
Read-length N50	12,984	18,415
Genomic coverage	57X	62X

The details of the resulting polished genomes are given in Table S2. Estimates of the fraction of the genomes covered by the assembly are based on genome size estimates

in Arnqvist et al. (2015). Genome completeness was assessed with BUSCO (v3.0.2b) (Simão et al. 2015), using the insecta_odb9 gene dataset. This showed a very high fraction of well-assembled genes in the assembly (Table S2).

**Table S2: Statistics for the genome assemblies.**		
	C. chinensis	***C. analis***
Nucletides (size, bp)	701,222,022	959,150,201
Sequences	4,563	12,242
GC content (%)	37.1	38.3
N50	800,721	240,251
Total BUSCO groups searched	1,658	1,658
Complete BUSCOs (%)	1,627 (98.1)	1,528 (92.1)
Complete single copy BUSCOs (%)	1,472 (88.8)	1,433 (86.4)
Complete duplicated BUSCOs (%)	155 (9.3)	95 (5.7)
Fragmented BUSCOs (%)	7 (0.4)	49 (3)
Missing BUSCOs (%)	24 (1.5)	81 (4.9)
Fraction of genome covered by assembly (%)	97.7	98.8

2. Genome annotation

The genome annotation service at the National Bioinformatics Infrastructure Sweden (www.nbis.se) carried out the genome annotation of *C. chinensis* and *C. analis*, using an identical comprehensive MAKER3 pipeline (Holt and Yandell, 2011) as detailed below. Protein sequence sets were collected for the annotations from the Uniprot Swiss-Prot database4 (downloaded on 2018-03; 556 825 proteins) and from two proteomes of the closely related seed beetle species *C. maculatus* (Sayadi et al. 2019) and *Acanthoscelides obtectus* (Immonen et al. 2023). We also used transcriptomes of these two species, in the form of a *de novo* assembly (*C. maculatus*; Sayadi et al. 2016) and 32 guided assemblies (*A. obtectus*; Immonen et al. 2023).

We first created species specific repeat libraries modeled using the RepeatModeler package (1.0.8) (Smit and Hubley 2010). All candidate repeats modeled by RepeatModeler were vetted against our protein sets (minus transposons) to exclude any nucleotide motif stemming from low-complexity coding sequences. From the repeat library, identification of repeat sequences present in the genome was then performed using RepeatMasker (4.0.3) (Smit et al. 2010) and RepeatRunner (Yandell 2006). In total 757,374 repeats were masked for *C. chinensis*, constituting a total size of 352,741 kb (50.3 % of the assembly) and 944,114 repeats were masked for *C. analis*, constituting a total size of 634,073 kb (66.1 % of the assembly).

A first round of annotation was performed with MAKER3 using both (1) curated protein sequences collected from the Uniprot Swiss-Prot database (Magrane 2011) and (2) the *de novo* assembly generated with Trinity (Grabherr et al. 2011) and the 32 guided assemblies made with StringTie (Pertea et al. 2015) of *C. maculatus* and *A. obtectus* mentioned above. This evidence-based gene builds resulted in a first "release candidate" gene set (rc1) with 27,601 gene models for *C. chinensis* and 24,345 gene models for *C. analis*.

The evidence-based annotation is limited by the available sequence data, which can lead to fragmented gene models and missed genes. To prevent this from happening, we next performed an *ab initio* evidence-driven gene build, where available protein and transcriptome evidence was used to help and guide *ab initio* tools during their prediction processes. From the first evidence-based gene build (rc1), we selected a high-confidence set of genes used to train the *ab initio* tools Augustus 2.7 (Stanke *et al.*, 2006) and Snap 2006-07-28 (Korf, 2004). We also trained GeneMark-ET 4.3 (Lomsadze *et al.*, 2014), which is a self-trained method integrating RNA-seq evidence using the *junctions.bed* file from Tophat. The *ab initio* evidence-driven annotation was performed with MAKER3, using both the output HMM-models from the trained *ab initio* tools (Augustus, Snap, and Genemark-ET) and the same evidence data as used previously. We also used EVidenceModeler (EVM) (Haas *et al.*, 2008), which allowed us to perform gene models based on the best possible set of exons produced by the other *ab initio* tools, and choose the most consistent according to the available

evidence. This *ab initio* evidence-driven gene builds (rc2) contained 34,872 gene models for *C. chinensis* and 32,965 gene models for *C. analis*.

Finally, all *ab initio* gene models (rc2) that mapped within an empty locus in the evidence-driven annotation (rc1), was added to rc1 to create our final build (rc3). The details of the annotations are given in Table S3.

**Table S3: Coding genes annotation statistics (rc3).**	***C. chinensis***	***C. analis***
Number of protein-coding genes	35,426	33,285
Number of mRNA	59,338	54,848
Average number of exons per mRNA	4.4	4.1
Average exon length	277	283
Average intron length	2,619	2,988
Average CDS length	1,051	1,042
Fraction of the genome covered by genes (%)	43.2	34.8

For the final gene build (rc3), we inferred putative functions for all genes. To this end, we first predicted functional domains using InterProscan 5.21-60 (Jones *et al.*, 2014) to retrieve functional information from Interpro (21 different sources). Functional annotations were thus assigned to 25,118 of the predicted coding genes and to 42,983 of the predicted mRNAs for *C. chinensis* and to 24,973 of the predicted coding genes and to 41,599 of the predicted mRNAs for *C. analis*. Each predicted protein sequence was also blasted against the Uniprot/Swissprot reference data set in order to infer, when available, the gene and protein name. The inference was made using the best blast hit approach, i.e. using the best hit with a maximum e-value cut-off to 1e-6. This made it possible to associate gene names to 15,014 genes for *C. chinensis* and to 13,648 genes for *C. analis*. In addition, 6,200 tRNA genes were

annotated for *C. chinensis* and 4,994 for *C. analis*, which were added to rc3, through *tRNAscan* 1.3.1 (Lowe and Eddy 1997).

The two genomes *de novo* assembled and annotated here, along with sequence data, is available from the European Nucleotide Archive (ENA) under the project ID PRJEB70760 for *C. chinensis* and the project ID PRJEB70763 for *C. analis*. The annotated genome assemblies for the two additional species used here were ENA accession PRJEB30475 for *C. maculatus* and PRJEB51445 for *A. obtectus*.

3. Orthogroup clustering

Based on data from Immonen et al. (2017) and Sayadi et al. (2019), we annotated four sets of genes for *C. maculatus* as digestive proteins (741), female reproductive proteins or FRPs (126), seminal fluid proteins or SFPs (185) and conserved single-copy genes (1137) across the Arthropod phylum. Next, we extracted the coding sequences of the proteomes for the three remaining beetle species and we identified hierarchical orthogroups among the four proteomes, using Orthofinder version 2.4 (Emms and Kelly, 2019). Nucleotide sequences where translated into amino acid sequences using transeq of the EMBOSS v6.6.0.0 package (Rice et al., 2000). A custom python script was used to extract the orthogroups which contained any *C. maculatus* protein that was annotated as digestive, SFP, FRP or conserved. A total of 88.7% of the total sum of genes belonging to all four species, was assigned into 28,673 hierarchical orthogroups, from which 1,129 contained conserved genes, 709 contained digestive proteins, 123 contained FRPs and 173 contained SFPs. To make sure that we retained orthologs only for the annotated *C. maculatus* proteins, we excluded all non-annotated *C. maculatus* genes from the orthogroups.

To align nucleotide sequences in each orthogroup, we employed a custom python script (backtranslate.py) to generate codon-aware alignments of CDS sequences. The script translated the CDS sequences into protein sequences using the Biopython translate() function, then used MAFFT v7.490 (Katoh et al., 2013) for protein alignments. Proteins were then back-translated into nucleotides. Fasttree version 2.1.11 (Price et al., 2010) was then used to make gene trees. Genes trees and

sequence alignments were given as inputs to phylopypruner v0.9.7 (Thalén et al., 2018) to extract strict orthogroups, i.e. orthologs only. Several prefiltering steps were used in phylopypruner to ensure a high quality of the strict orthogroups. These were the removal of (i) any sequence below 100 base pairs, (ii) any sequence with a branch length longer than five times the standard deviation of all branches within a hierarchical orthogroup, so as to avoid long branch attraction, (iii) any sequences that belong to different taxa, but have very short branch lengths and (iv) any sequence with support value below 70% on the gene tree. We used *A. obtectus* as the outgroup and paralogy pruning was done using the maximum inclusion method. Postfiltering steps via phylopypruner required the minimum taxa allowed being equal to four, the forced inclusion of *C. maculatus* sequences in the orthogroups and removal of any sequence with gaps occupying more than 20% of the alignment length. From the 1,129 orthogroups with conserved Busco genes, 792 passed the filtering criteria and were converted to strict orthogroups. Corresponding numbers were 709 and 337 for digestive proteins, 123 and 55 for FRPs and 173 and 68 for SFPs. Missing data (gaps in alignments) varied from 11.6% to 29.9% before filtering and from 4.0% to 5.9% after filtering.

4. Estimation of dN:dS ratios

We used codeML 4.8a of the PAML package (Yang 2007) via the ETE3 python framework (Huerta-Cepas et al., 2016). For all strict orthogroups, we run the sites models M0, M1a, M2a, M7, M8 and M8a. In addition, we fitted free-ratios branch models. These were run four times for each strict orthogroup, each time with a different species as the foreground branch and the rest as the background branches. We performed likelihood ratio tests (LRTs) between pairs of models to test for signs of neutrality and positive selection across sites and/or across species. The LRTs were based on the M1a-M2a, M7-M8 and the M8-M8a model comparisons. The “cleandata” parameter was set to 1, in order to remove gaps and ambiguity characters from the alignments. Python scripts were used to extract omega values for each model and the likelihood ratios for each LRT.

5. Evolutionary Rate Covariation

For each strict orthogroup, we first estimated branch specific omega based on free-ratios models, assuming that protein sites evolve with one common rate which may differ between species. For all 530 orthogroups, we then performed all pairwise correlations between the logarithm of branch-specific omega values. The logarithm was used in order to normalize the distribution of the omega values. We also assessed FDR corrected p-values for the Pearson's r correlations for the SFP-FRP set, which highlighted five pairs of evolutionary rate covariation (Table S4). One SFP showed correlated evolution with three different FRPs .

Table S4. Pairs of SFPs and FRPs showing significantly correlated evolution (gene names refer to the *C. maculatus* reference genome).

SFP	FRP	Pearson's r
CALMACT00000035026	CALMACT00000004474	0.998
CALMACT00000016355	CALMACT00000031120	0.998
CALMACT00000024208	CALMACT00000010734	0.999
CALMACT00000024208	CALMACT00000014632	0.999
CALMACT00000024208	CALMACT00000022367	1.00

6. McDonald – Kreitman tests

As a source of within-species nucleotide polymorphism, we used data from Sayadi et al (2019) consisting of pool-seq re-sequencing of a population of *C. maculatus* (Brazil). Variants were called based on the reference genome of *C. maculatus* (accession PRJEB30475). Pool-seq read data were first aligned on the reference genome using bowtie2 v2.5.2 and samtools mpileup (v1.4 with parameters -B -d 1000 -t DP,AD,ADF,ADR -uf). We then used bcftools call (v1.3 with parameters -cv) to call variants. SNP filtering was done using bcftools view -i 'QUAL>30 & INFO/DP>10 & MQ>30 & INFO/AF>0.05', to remove low quality variants. The effect of SNPs were identified using SNPEff v5.2a, thus identifying the number of non-synonymous (pN) and synonymous sites (pS) within genes. McDonald-Kreitman tests were then done by calculating the Neutrality Index (NI) (Rand and Kann 1996), as (pN/pS)/(dN/dS). Fisher's exact tests were performed to get significance levels of deviation of NI from

1, by using the fisher.test() function in R, which were then FDR compensated ($q < 0.05$).

7. Linear models

We created a linear model to link gene expression, sex-biased expression and protein type to the overall omega values (from the M0 model). In this model, protein type was included as a categorical factor, level of gene expression as the logarithm of fragments per kilobase of transcript per million of mapped reads (FPKM from now on) and the sex-bias in expression (LOGFC from now on). Data on gene expression was retrieved from the study of Immonen et al. (2017) of *C. maculatus*.

Data on FPKM and LOGFC was standardized, by subtracting their mean and dividing by their standard deviation, prior to modelling. To normalize residuals of our inferential model, we transformed omega values using the one-parameter Boxcox function of the MASS package in R (yielding an optimal $\lambda = 0.303$). Our model included as explanatory variables the protein type, FPKM, LOGFC and all two-way interaction terms (see Table 1). A multiple regression model including only FPKM, LOGFC and their interaction yielded an R^2-value of 0.2, suggesting that these variables alone explain approximately 20% of the variance in omega values.

Because data on gene expression was collected in *C. maculatus*, we also replicated these efforts but instead using omega values from the *C. maculatus* branch in free-ratios models. These analyses yielded results that were qualitatively identical to those reported with omega values from M0 models (Figure S1).

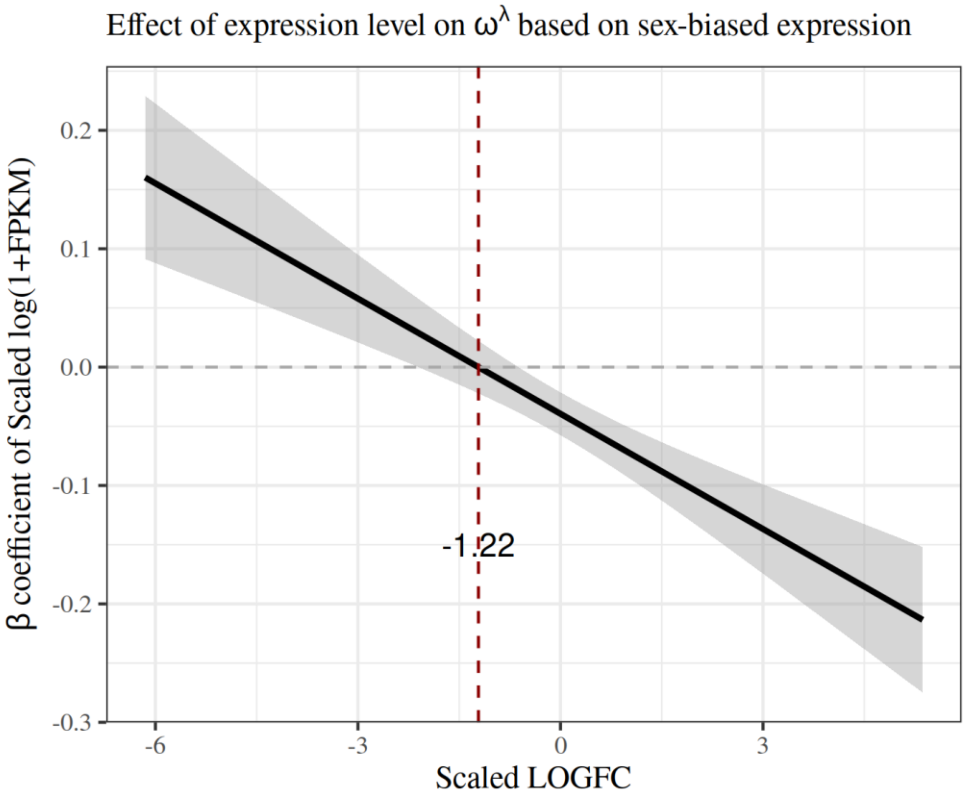

Figure S1. The relationship between overall gene expression and ω is negative for genes with female-biased expression and those with unbiased expression but postitive for genes with male-biased expression. This figure visualizes the interactive effect of FPKM:LogFC on ω, where ω was estimated from the *C. maculatus* branch in free-ratios branch models (see Figure 5 for an analogous figure where ω was instead estimated from M0 models). The figure shows the predicted conditional effect of sex-biased expression (LogFC) along the abscissa on the slope (± 95% CI) between overall gene expression (log FPKM) and ω, along the ordinate. Genes with negative LogFC values are male-biased and those with positive values are female-biased. We note that the slope here is, if anything, steeper than for ω estimated from M0 models (cf. Figure 5).

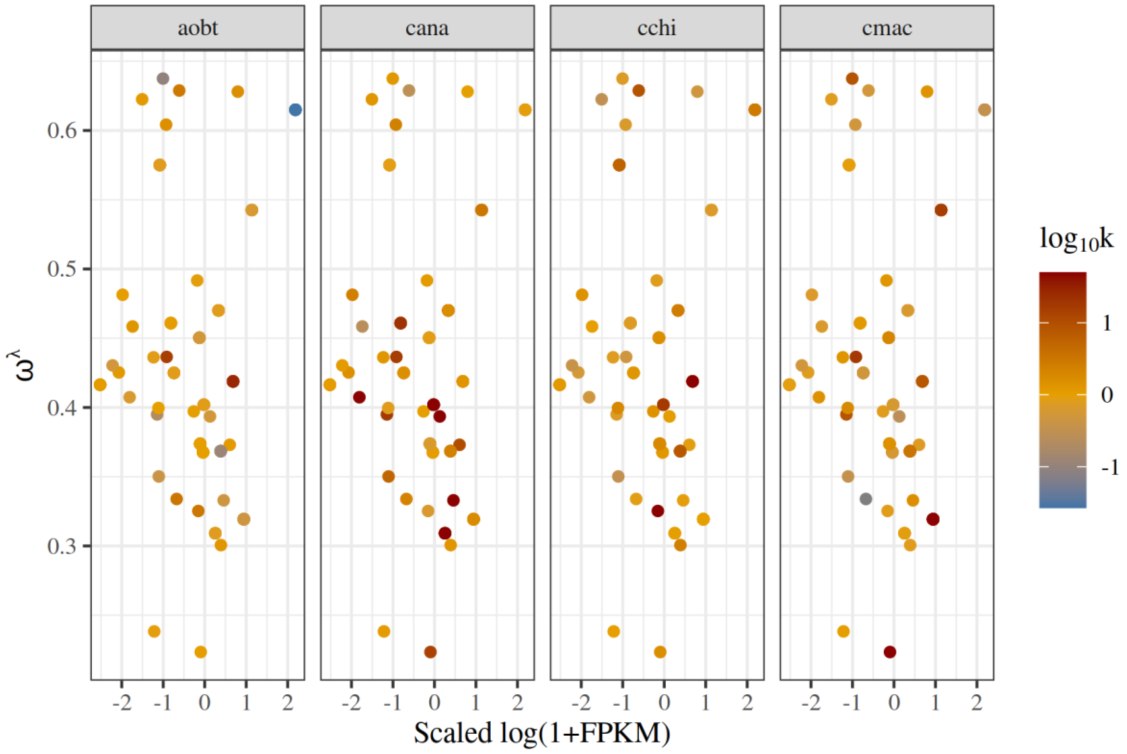

Figure S2. This figure shows ω estimated from M0 models plotted against gene expression for SFPs only (cf. Figure 4 for all genes). The E-R anticorrelation is nullified for SFPs, and symbols in this plot are colored based on $\log_{10}$ of the estimate of the k parameter from RELAX (using either of the four species as the foreground branch). Here, blue proteins are those showing signs of relaxed selection (k<1) and red symbols are those showing signs of intensified selection (k>1). A visual inspection suggests that the E-R anticorrelation is not nullified by highly expressed SFPs with a high ω (upper-right quadrant) generally experiencing relaxed selection. While one such protein shows signs for relaxed selection along the *A. obtectus* branch, most do not and several others if anything instead show intensified selection along other branches.

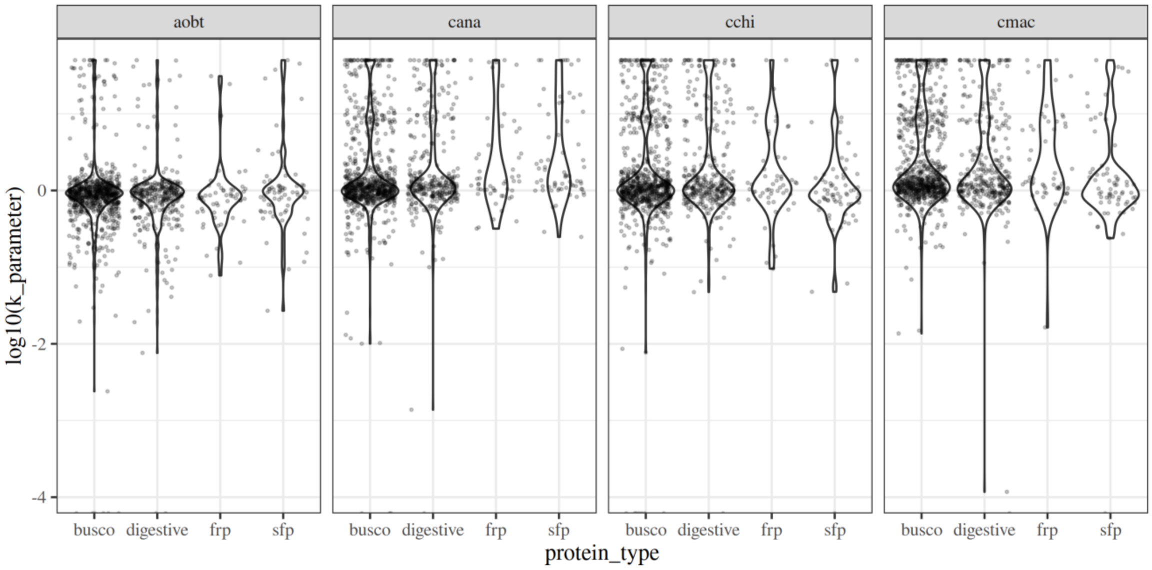

Figure S3. Violin plots of $\log_{10}$ of the estimate of the k parameter from RELAX, using either of the four species as the foreground branch, for the four classes of proteins. Proteins showing signs of relaxed selection (k<1) have negative values and those showing signs of intensified selection (k>1) have positive values on this scale. The proportion of proteins showing significantly relaxed selection was low and did not differ significantly across protein types.

8. Phylogeny

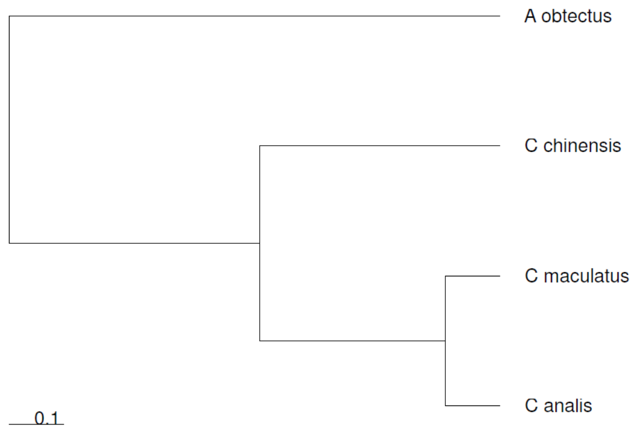

Figure S4. Phylogenetic relationship between the four species used here.

9. References

Arnqvist G, Sayadi A, Immonen E, Hotzy C, Rankin D, Tuda M, Hjelmen CE, Johnston JS. 2015. Genome size correlates with reproductive fitness in seed beetles. Proc. R. Soc. B. 282:20151421. DOI: 10.1098/rspb.2015.1421.

Emms DM, Kelly S. 2019. OrthoFinder: Phylogenetic Orthology Inference for Comparative Genomics. Genome Biol. 20:238. DOI: 10.1186/s13059-019-1832-y.

Grabherr MG, Haas BJ, Yassour M, Levin JZ, Thompson DA, Amit I, Adiconis X, Fan L, Raychowdhury R, Zeng Q, et al. 2011. Full-length transcriptome assembly from RNA-Seq data without a reference genome. Nat. Biotechnol. 29:644-652. DOI: 10.1038/nbt.1883.

Haas BJ, Salzberg SL, Zhu W, Pertea M, Allen JE, Orvis J, White O, Buell CR, Wortman JR. 2008. Automated eukaryotic gene structure annotation using EVidenceModeler and the Program to Assemble Spliced Alignments. Genome Biol. 9:R7. DOI: 10.1186/gb-2008-9-1-r7.

Holt C, Yandell M. 2011. MAKER2: an annotation pipeline and genomedatabase management tool for second-generation genome projects. BMC Bioinformatics. 12:491. DOI: 10.1186/1471-2105-12-491.

Huerta-Cepas J, Serra F, Bork P. 2016. ETE 3: Reconstruction, Analysis, and Visualization of Phylogenomic Data. Mol. Biol. Evol. 33:1635-1638. DOI: 10.1093/molbev/msw046.

Immonen E, Sayadi A, Bayram H, Arnqvist G. 2017. Mating Changes Sexually Dimorphic Gene Expression in the Seed Beetle Callosobruchus maculatus. Genome Biol. Evol. 9:677-699. DOI: 10.1093/gbe/evx029.

Immonen E, Sayadi A, Stojković B, Savković U, Đorđević M, Liljestrand-Rönn J, Wiberg RA, Arnqvist G. 2023. Experimental life history evolution results in sex-specific evolution of gene expression in seed beetles. Genome Biol. Evol. 15, evac177.

Jones P, Binns D, Chang HY, Fraser M, Li W, McAnulla C, McWilliam H, Maslen J, Mitchell A, Nuka G, et al. 2014. InterProScan 5: genome-scale protein function classification. Bioinformatics. 30:1236-1240. DOI: 10.1093/bioinformatics/btu031.

Katoh K, Standley DM. 2013. MAFFT Multiple Sequence Alignment Software Version 7: Improvements in Performance and Usability. Mol. Biol. Evol. 30:772-780. DOI: 10.1093/molbev/mst010.

Korf I. 2004. Gene finding in novel genomes. BMC Bioinformatics. 5:59. DOI: 10.1186/1471-2105-5-59.

Lomsadze A, Burns PD, Borodovsky M. 2014. Integration of mapped RNA-Seq reads into automatic training of eukaryotic gene finding algorithm. Nucleic Acids Res. 42:e119. DOI: 10.1093/nar/gku557.

Lowe TM, Eddy SR. 1997. tRNAscan-SE: A Program for Improved Detection of Transfer RNA Genes in Genomic Sequence. Nucleic Acids Res. 25:0955-0964. DOI: 10.1093/nar/25.5.0955.

Magrane M, UniProt Consortium. 2011. UniProt Knowledgebase: a hub of integrated protein data. Database (Oxford). 2011:bar009. DOI: 10.1093/database/bar009.

Pertea M, Pertea GM, Antonescu CM, Chang TC, Mendell JT, Salzberg SL. 2015. StringTie enables improved reconstruction of a transcriptome from RNA-seq reads. Nat. Biotechnol. 33:290-295. DOI: 10.1038/nbt.3122.

Price MN, Dehal PS, Arkin AP. 2010. FastTree 2 – Approximately Maximum-Likelihood Trees for Large Alignments. PLoS ONE. 5:e9490. DOI: 10.1371/journal.pone.0009490.

Rand DM, Kann LM. 1996. Excess Amino Acid Polymorphism in Mitochondrial DNA: Contrasts among Genes from Drosophila, Mice, and Humans. Mol. Biol. Evol. 13:735-748. DOI: 10.1093/oxfordjournals.molbev.a025634.

Rice P, Longden I, Bleasby A. 2000. EMBOSS: The European Molecular Biology Open Software Suite. Trends Genet. 16:276-277. DOI: 10.1016/s0168-9525(00)02024-2.

Sayadi A, Immonen E, Bayram H, Arnqvist G. 2016. The de novo transcriptome and its functional annotation in the seed beetle Callosobruchus maculatus. PLoS ONE. 11:e0158565. DOI: 10.1371/journal.pone.0158565.

Sayadi A, Martinez Barrio A, Immonen E, Dainat J, Berger D, Tellgren-Roth C, Nystedt B, Arnqvist G. 2019. The genomic footprint of sexual conflict. Nat. Ecol. Evol. 3:1725-1730. DOI: 10.1038/s41559-019-1034-3.

Simão FA, Waterhouse RM, Ioannidis P, Kriventseva EV, Zdobnov EM. 2015. BUSCO: assessing genome assembly and annotation completeness with single-copy orthologs. Bioinformatics. 31:3210-3212. DOI: 10.1093/bioinformatics/btv351.

Smit A, Hubley R, Green P. 2010. RepeatMasker Open-3.0. Available from: http://www.repeatmasker.org.

Smit A, Hubley R. 2010. RepeatModeler Open-1.0. Available from: http://www.repeatmasker.org.

Stanke M, Schöffmann O, Morgenstern B, Waack S. 2006. AUGUSTUS: ab initio prediction of alternative transcripts. Nucleic Acids Res. 34:435-439. DOI: 10.1093/nar/gkl200.

Thalén F. 2018. PhyloPyPruner: Tree-Based Orthology Inference for Phylogenomics with New Methods for Identifying and Excluding Contamination. MSc thesis, Lund University. Available from: http://lup.lub.lu.se/student-papers/record/8963554.

Yandell M. 2006. Comparative Genomics Library — RepeatRunner. Available from: http://www.yandell-lab.org.

Yang Z. 2007. PAML 4: Phylogenetic Analysis by Maximum Likelihood. Mol. Biol. Evol. 24:1586-1591. DOI: 10.1093/molbev/msm088.
